# Pactacin is a novel digestive enzyme in teleosts

**DOI:** 10.1038/s41598-021-86565-9

**Published:** 2021-03-31

**Authors:** Mari Kawaguchi, Yohei Okazawa, Aiko Imafuku, Yuko Nakano, Risa Shimizu, Reiji Ishizuka, Tianlong Jiang, Tatsuki Nagasawa, Junya Hiroi, Shigeki Yasumasu

**Affiliations:** 1 Department of Materials and Life Sciences, Faculty of Science and Technology, Sophia University, 7-1 Kioi-cho, Chiyoda-ku, Tokyo, 102-8554 Japan; 2 Department of Life Science and Technology, Tokyo Institute of Technology, 2-12-1 Ookayama, Meguro-ku, Tokyo, 152-8550 Japan; 3 Department of Anatomy, St. Marianna University School of Medicine, 2-16-1 Sugao, Miyamae-ku, Kawasaki, 216-8511 Japan; 4 Present Address: Department of Biochemistry and Molecular Biology, Thomas Jefferson University, 233 South 10th Street, 220 BLSB, Philadelphia, PA 19107 USA

**Keywords:** Proteases, Zoology

## Abstract

Generally, animals extract nutrients from food by degradation using digestive enzymes. Trypsin and chymotrypsin, one of the major digestive enzymes in vertebrates, are pancreatic proenzymes secreted into the intestines. In this investigation, we report the identification of a digestive teleost enzyme, a pancreatic astacin that we termed pactacin. Pactacin, which belongs to the astacin metalloprotease family, emerged during the evolution of teleosts through gene duplication of astacin family enzymes containing six cysteine residues (C6astacin, or C6AST). In this study, we first cloned C6AST genes from pot-bellied seahorse (*Hippocampus abdominalis*) and analyzed their phylogenetic relationships using over 100 C6AST genes. Nearly all these genes belong to one of three clades: pactacin, nephrosin, and patristacin. Genes of the pactacin clade were further divided into three subclades. To compare the localization and functions of the three pactacin subclades, we studied pactacin enzymes in pot-bellied seahorse and medaka (*Oryzias latipes*). In situ hybridization revealed that genes of all three subclades were commonly expressed in the pancreas. Western blot analysis indicated storage of pactacin pro-enzyme form in the pancreas, and conversion to the active forms in the intestine. Finally, we partially purified the pactacin from digestive fluid, and found that pactacin is novel digestive enzyme that is specific in teleosts.

## Introduction

Astacin family proteases are members of the metalloendopeptidases, showing a variety of functions, such as developmental regulation (bone morphogenetic protein 1; BMP1), digestive proteolysis (astacin), and processing of extracellular proteins (meprin)^1,2^. These proteases are synthesized as pre-proenzymes consisting of a signal peptide, a proenzyme region, and an astacin protease domain. Some of these proteases possess multiple domains apart from the astacin protease domain. Four cysteine residues are located in the astacin protease domain of all astacin proteases. In addition, two cysteine residues are present in some astacin family metalloproteases, called six-cysteine-containing astacins (C6astacin, abbreviated to C6AST)^3^. These C6ASTs include hatching enzymes in vertebrates, and nephrosin and patristacin in fish. Hatching enzymes have been studied extensively, mainly using medaka (*Oryzias latipes*)^4,5^. These enzymes digest the egg envelope at the time of hatching. In contrast, the functions of other C6ASTs, such as nephrosin and patristacin, have so far remained elusive.

The nephrosin gene was first discovered in the head kidney, trunk kidney, and spleen of carp (*Cyprinus carpio*)^6^. In 2017, using the CRISPR/Cas9 system in zebrafish (*Danio rerio*), nephrosin-deficient mutants were obtained to investigate the function of nephrosin^7^. Despite having the same neutrophil number as non-mutated individuals, mutant zebrafish exhibited an increased inflammatory response and a lower survival rate on infection with *Escherichia coli*. Conversely, overexpression of nephrosin enhanced host defense against *E. coli* infections. These results suggest that nephrosin promotes protection against bacterial infections^7^.

Patristacin was first discovered in gulf pipefish (*Syngnathus scovelli*)^8^. Pipefishes, together with seahorses (*Hippocampus*), are syngnathiform fishes, which uniquely possess a brood pouch in males, used for embryo incubation^9^. It was shown that the patristacin gene is expressed in the brood pouch of gulf pipefish, and five copies of it were located on the same chromosome^10^. Two of the five gene copies exhibited pronounced expression related to pregnancy, one in the pregnant state and the other in the non-pregnant state. Similarly, from tiger tail seahorse (*Hippocampus comes*), six copies of the patristacin gene were identified, the expression of five of which was enhanced during mid- and late pregnancy, but suppressed in non-pregnant and post parturition tissue^11^. These results suggest that the function of the patristacin gene is common in syngnathid species, and is speculated, but not yet proved, to involve regulation of hatching within the brood pouch^12^.

We previously surveyed genome databases and found five C6AST gene homologs in medaka, called MC6AST1–5^3^. MC6AST1 corresponded to nephrosin, MC6AST2 and MC6AST3 showed the same expression (mainly in the mesentery), and MC6AST4 and MC6AST5 were both expressed in the jaw. Patristacin gene orthologs were not found from the medaka genome. In the present study, we first focused on nephrosin, patristacin, and MC6AST2–3, because these gene orthologs seem to have shared synteny at some point of evolution before separating into different chromosomes, suggesting a close evolutionary relationship. We cloned the three types of gene orthologs from pot-bellied seahorse and performed phylogenetic analysis to elucidate their evolutional relationships. Among the three gene orthologs, MC6AST2–3 genes have to date received the least attention. To investigate the functions of the MC6AST2–3 genes, we analyzed their expression in seahorse and medaka. We observed that MC6AST2–3 gene orthologs are commonly expressed in pancreatic cells, and consequently named them pactacin (pancreatic astacin) genes.

## Results

### Cloning of C6AST genes from seahorse

**Figure 1:**
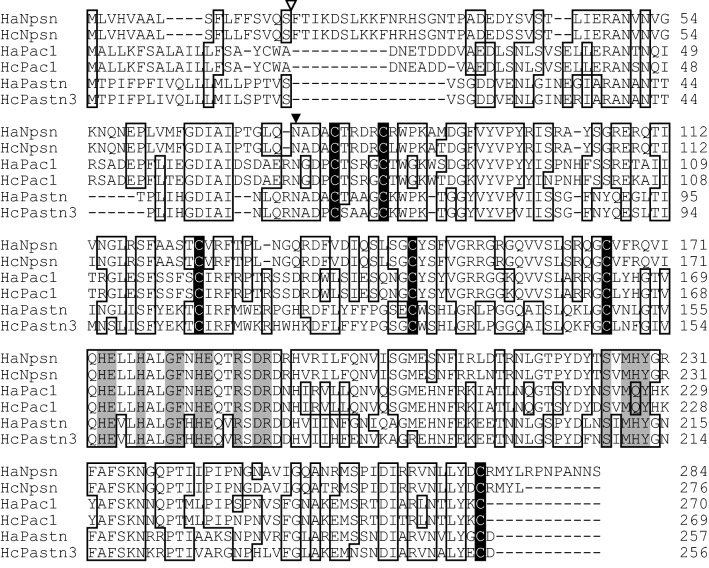
Multiple alignment of amino acid sequences of nephrosin, pactacin and patristacin of pot-bellied seahorse (HaNpsn, HaPac1 and HaPastn) and tiger tail seahorse (HcNpsn, HcPac1 and HcPastn3). Identical residues are boxed. Dashes represent gaps. Two active site consensus sequences of the astacin family proteases (Zn-binding site, HExxHxxGFxHExxRxDR, and methionine turn, SxMHY) are indicated in gray boxes. Conserved cysteine residues are shaded in black. Open and solid triangles indicate putative signal sequence cleavage sites and N-termini of the astacin protease domain, respectively.

Using the degenerate primers, three kinds of full-length C6AST genes were amplified by RT-PCR from RNAs of pot-bellied seahorse. Based on the phylogenetic analysis described below, we called these genes HaNpsn (cloned from the kidney), HaPac1 (cloned from the liver) and HaPastn (cloned from the brood pouch). Their deduced amino acid sequences contain the signal sequence, pro-sequence, and the astacin protease domain, in which the active-site consensus sequence of astacin family proteases and six cysteine residues are conserved (Fig. 1).

### Phylogenetic analysis of C6AST genes

**Figure 2:**
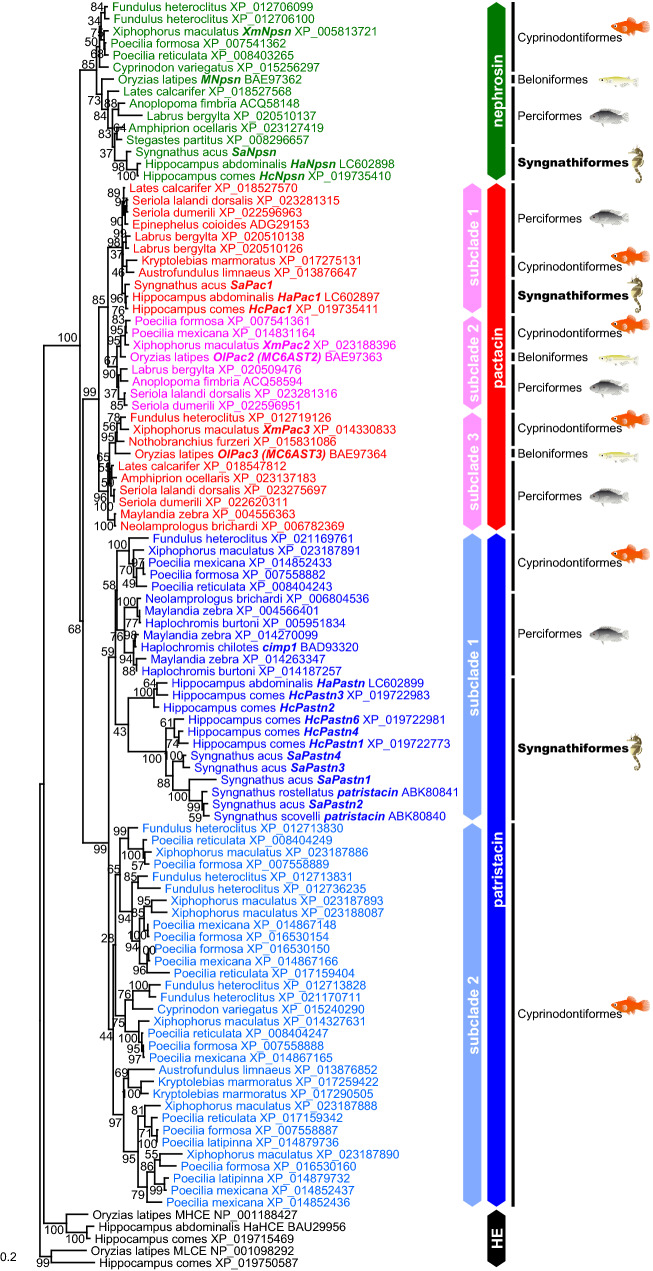
Maximum-likelihood tree of C6AST genes. Green, red, and blue indicate genes for nephrosin, pactacin, and patristacin, respectively. Gene names described in the present study are italicized. Node numbers indicate bootstrap values, shown as percentages. GenBank accession numbers of sequences are shown to the right of the species name. HE = hatching enzyme.

Homologous genes of the HaNpsn, HaPac1 and HaPastn were searched in the GenBank database. In total, 100 homologous genes, including seven homologous genes of tiger tail seahorse (HcNpsn XP_019735410, HcPac1 XP_019735411, HcPastn1 XP_019722773, HcPastn2, HcPastn3 XP_019722983, HcPastn4, HcPastn6 XP_019722981) and six homologous genes of greater pipefish (SaNpsn, SaPac1, SaPastn1, SaPastn2, SaPastn3, and SaPastn4) were found. Using these genes, maximum-likelihood analysis was performed to construct the phylogenetic tree, using five hatching enzyme genes as an outgroup (Fig. 2). The tree is primarily divided into three clades, namely, nephrosin, pactacin, and patristacin.

The cloned HaNpsn, tiger tail seahorse HcNpsn and greater pipefish SaNpsn genes and medaka MNpsn gene, which is known to be expressed mainly in the kidney^3^, are located in the nephrosin clade.

The cloned HaPastn, tiger tail seahorse HcPastn1–4 and 6, and greater pipefish SaPastn1–4 genes are located in the patristacin clade. This clade is further divided into two subclades. Subclade 1 is composed of genes from various teleostean fishes, such as cyprinodontiform, perciform, and syngnathiform fishes. Seahorse and pipefish patristacin genes located in subclade 1 are both expressed in the brood pouch, as reported previously^8^. Therefore, HaPastn is orthologous to the patristacin gene. On the other hand, subclade 2 genes are only found in cyprinodontiform fishes.

The cloned HaPac1, tiger tail seahorse HcPac1, and greater pipefish SaPac1 genes are located in the pactacin clade. This clade is further divided into three subclades, and the HaPac1 gene is located in subclade 1. In contrast, MC6AST2 and MC6AST3 are located in subclades 2 and 3, respectively, and were previously reported to be expressed in the mesenterium in medaka^3^. As described below, HaPac1, MC6AST2 and MC6AST3 are expressed primarily in the pancreas. Therefore, we renamed MC6AST2–3 as OlPac2–3.

### Synteny

Synteny of the C6AST genes was examined using the genome databases of tiger tail seahorse, greater pipefish, medaka and platyfish (*Xiphophorus maculatus*).

**Figure 3:**
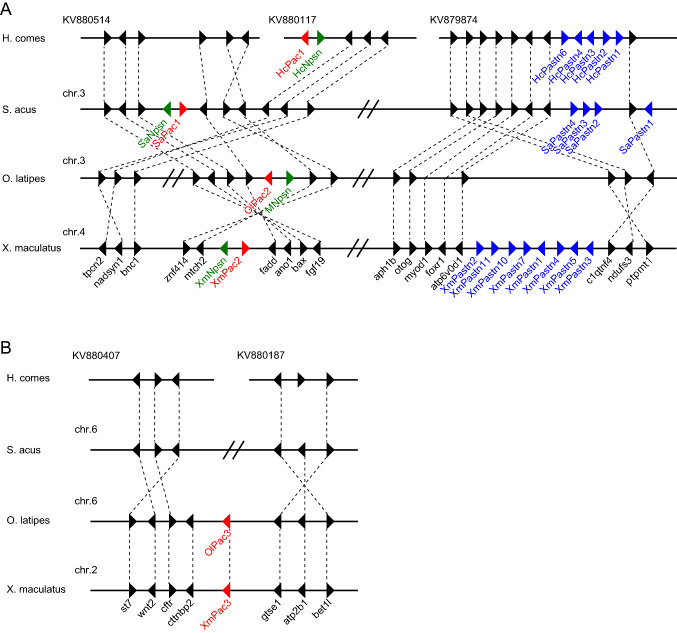
Synteny of genomic regions around (**A**) nephrosin, pactacin (subclades 1 and 2) and patristacin genes, and (**B**) the pactacin (subclade 3) gene of tiger tail seahorse, greater pipefish, medaka, and platyfish.

In tiger tail seahorse, both HcNpsn and HcPac1 were found in contig KV880117 (Fig. 3A). HcPac1 is located at the end of the contig, and HcNpsn is located next to the tpcn2 gene. Five copies of HaPastn gene orthologs (HcPastn1, 2, 3, 4, and 6) were found in contig KV879874 (Fig. 3A). According to Lin et al., six copies of patristacin genes (pastn1–6) are present in scaffold 2 of tiger tail seahorse^11^, but we could find only five copies. Because of this discrepancy, the gene names used herein do not correspond to those used by Lin et al. For greater pipefish, SaPastn1–4, SaPac1, and SaNpsn genes are located on chromosome 3. This gene synteny is conserved in both tiger tail seahorse and greater pipefish (Fig. 3A).

One copy of nephrosin (XmNpsn), two copies of pactacin in each of subclades 2 and 3 (XmPac2 and XmPac3), and eight copies of patristacin (XmPastn1–5, 7, 10 and 11) were found in platyfish. All these genes are located on chromosome 4, except the XmPac3 gene, which is found on chromosome 2 (Fig. 3B). These gene locations are well conserved in medaka; in particular, OlPac2 and MNpsn are both located on chromosome 3, while OlPac3 is located on chromosome 6.

Summing up the results, C6AST gene synteny is conserved among the four species studied. Focusing on pactacin genes, genes of subclade 1 and 2 share synteny, while subclade 3 genes are located on different chromosomes. We next present a more detailed investigation of the HaPac1, OlPac2 (MC6AST2) and OlPac3 (MC6AST3) genes to compare the localization and function of the three pactacin subclade genes.

### Expression analysis of pactacin genes

**Figure 4:**
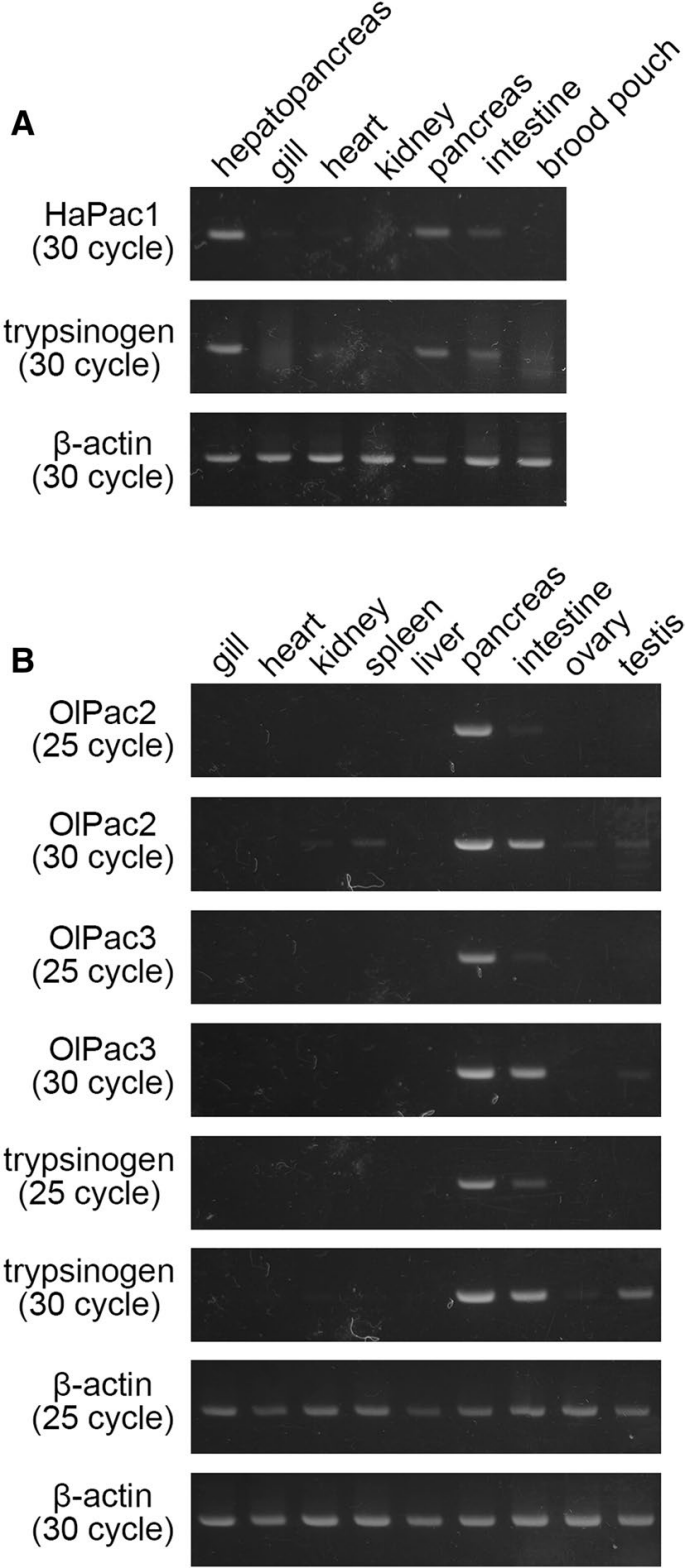
Semi-quantitative expression analysis of pactacin and trypsinogen genes. (**A**) HaPac1 and trypsinogen gene expression in adult seahorse hepatopancreas, gills, heart, kidney, mesentery including pancreas (labeled as pancreas), intestines, and brood pouch. (**B**) OlPac2 (MC6AST2), OlPac3 (MC6AST3), and trypsinogen gene expression in adult medaka gills, heart, kidney, spleen, liver, mesentery including pancreas (labeled as pancreas), intestines, ovary and testes. β-actin was used as a control signal. Band regions are cropped, and full-length gels are included in a Supplementary Information.

The expression pattern of pactacin genes was first analyzed by RT-PCR using RNAs extracted from adult seahorse and medaka. For seahorse, the strongest HaPac1 bands are observed in the hepatopancreas and mesentery, including the pancreas, and a weaker band is observed in the intestines (Fig. 4A). In medaka, both the OlPac2 (MC6AST2) and OlPac3 (MC6AST3) genes are expressed strongly in the mesentery, including the pancreas, and weakly in the intestines (Fig. 4B). These results indicate that pactacin genes are expressed in pancreatic tissues.

Pancreatic tissue is known to synthesize digestive enzymes, such as trypsin. As a control experiment, we further examined the expression of the trypsinogen gene (accession number: NM_001104900), the precursor of trypsin, by RT-PCR. The expression pattern of the trypsinogen gene in seahorse is the same as that of the HaPac1 gene, i.e., strong bands are observed in the hepatopancreas and the mesentery, including the pancreas, and a weak band is found in the intestines (Fig. 4A). Similarly, in medaka, the trypsinogen gene is expressed primarily in the mesentery, including the pancreas, and weakly in the intestines and testes. These results agree with a previous report^13^. It can be concluded that gene expression of the three pactacins corresponds closely to that of trypsinogen genes in seahorse and medaka.

### Localization of pactacin

**Figure 5:**
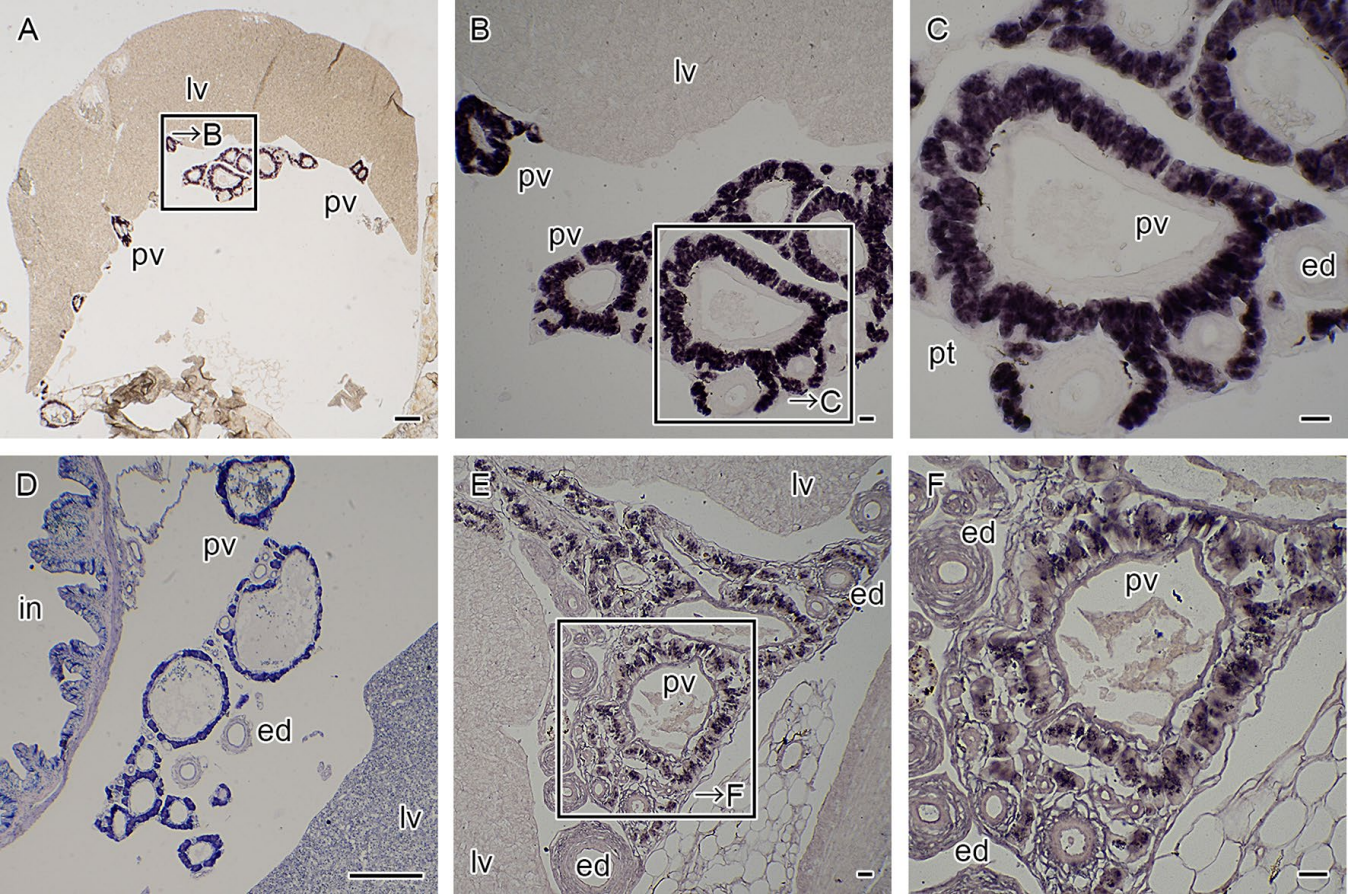
Localization of HaPac1 in pot-bellied seahorse. Organs were hybridized with HaPac1-antisense probe (**A**–**C**), stained with hematoxylin–eosin (**D**) to identify the portal veins (pv) and extrahepatic bile duct (ed), or immunostained with anti-HaPac1 antibody (**E**,**F**). The lettered boxes in (**A**), (**B**), and (**E**) indicate sites of high magnification views shown in (**B**), (**C**), and (**F**), respectively. Positive signals were stained purple. ed, extrahepatic bile duct; in, intestine; lv, liver; pt, pancreatic tissue; pv, portal vein. Scale bars: (**A**), (**D**) = 200 μm; (**B**), (**C**), (**E**), (**F**) = 20 μm.

Localization of the seahorse HaPac1 gene was first analyzed by in situ hybridization using the liver, pancreas, intestines, and kidney. Euteleostean pancreatic tissues are often scattered around the liver and surround portal veins, which enter from the intestines into the liver (Fig. 5D)^14^. The antisense probe of HaPac1 gene detected positive signals from pancreatic cells surrounding the portal veins and extrahepatic bile duct, but not from hepatic cells or intestines (Fig. 5A–C). Immunohistochemistry tests utilizing anti-HaPac1 antibodies revealed the presence of the seahorse HaPac1 protein in granules of the pancreatic cells, which are attached to portal veins and the extrahepatic bile duct (Fig. 5E,F). Thus, the localization of the HaPac1 protein corresponds closely to tissues in which the HaPac1 gene is expressed.

**Figure 6:**
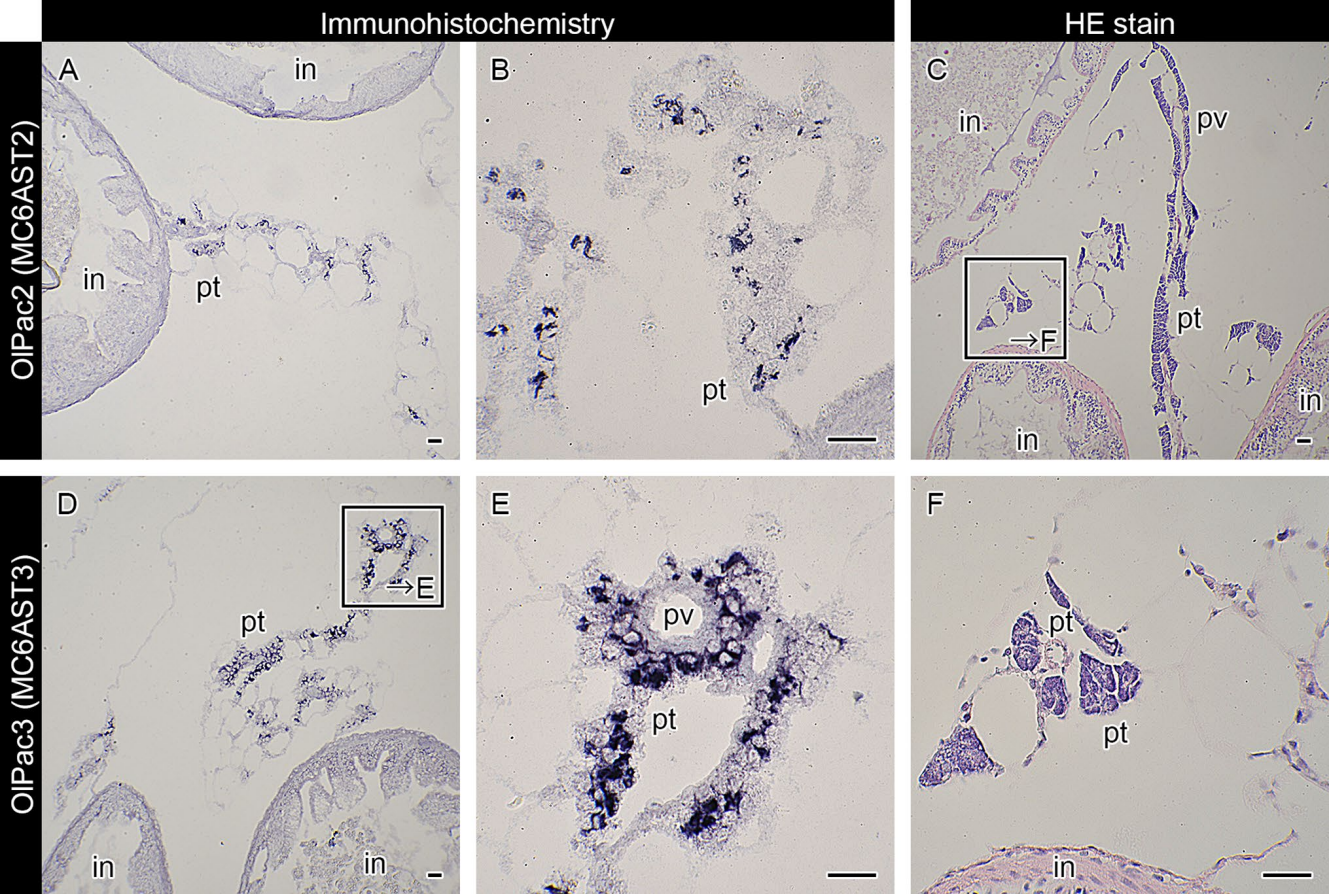
Localization of OlPac2 (MC6AST2) and OlPac3 (MC6AST3) in medaka. Organs were immunostained with anti-OlPac2 (**A**,**B**) or anti-OlPac3 antibody (**D**,**E**). The lettered boxes in (**C**,**D**) indicate sites of high magnification views shown in (**F**,**E**), respectively. Positive signals were stained purple. Organs were stained with hematoxylin–eosin (**C**,**F**) to identify the portal veins (pv). in, intestine; pt, pancreatic tissue; pv, portal veins. Scale bars: 20 μm.

In medaka, immunohistochemistry using anti-OlPac2 and anti-OlPac3 antibodies revealed the presence of both OlPac2 and OlPac3 in pancreatic cells around portal veins in the mesentery (Fig. 6A,B,D,E). It is reported that pancreatic tissues are divided into exocrine and endocrine components in medaka, and exocrine cells are present in the mesentery^15^, which connects to the gut (Fig. 6C,F). According to an older study, medaka OlPac2 (MC6AST2) and OlPac3 (MC6AST3) genes are expressed in the mesentery^3^. The present results are consistent with both these studies, i.e., we observed that pactacin genes in subclades 1–3 are expressed in pancreatic cells.

### Western blot analysis

**Figure 7:**
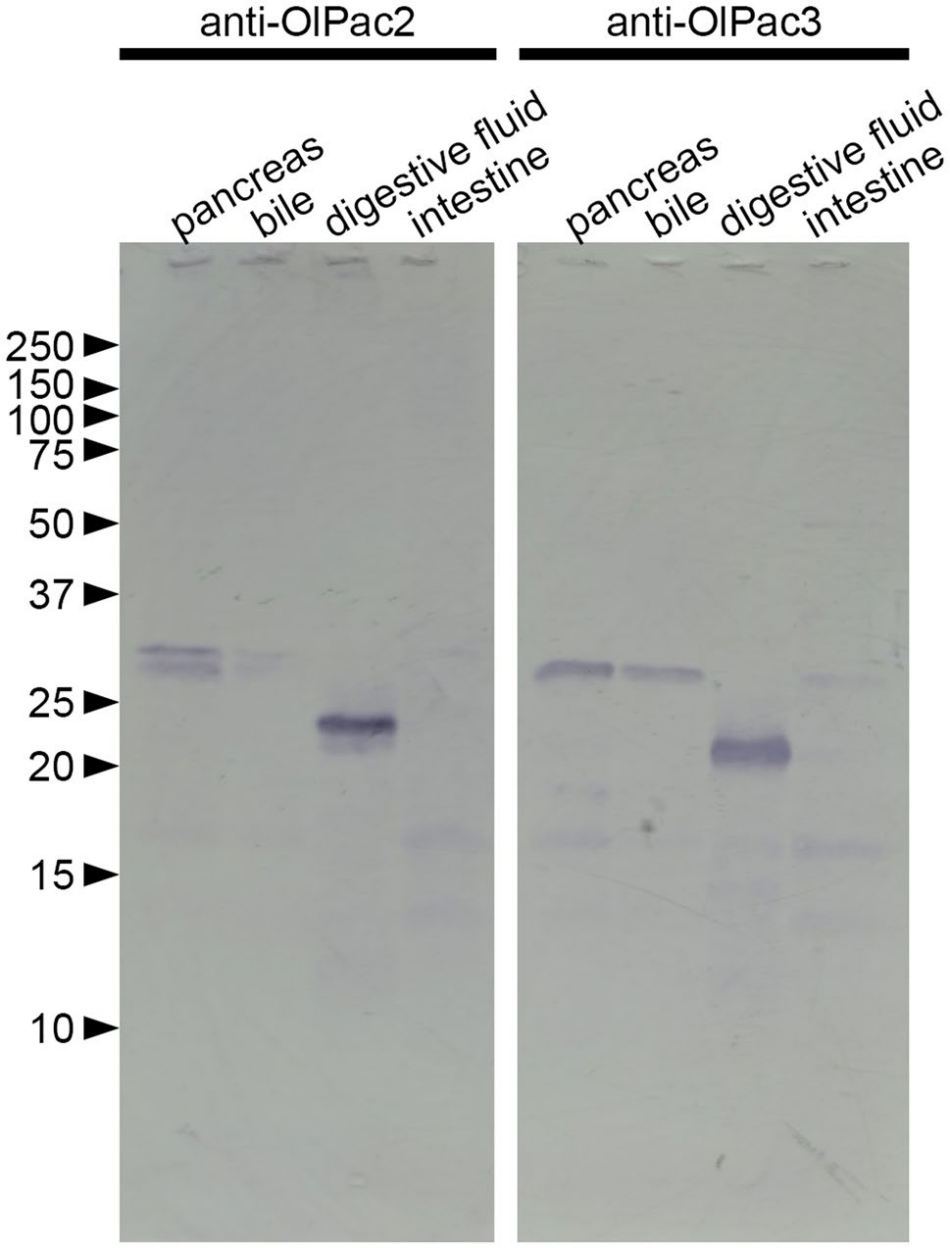
Western blot analysis of pactacin. Medaka extracts of the mesentery including pancreas (labeled as pancreas), intestines, bile and digestive fluid with anti-OlPac2 antibody or anti-OlPac3 antibody. Numbers on the left refer to molecular weights (kDa) of molecular markers. Full-length blots are shown, and original figures including marker lane are included in a Supplementary Information.

We carried out western blot analysis using extracts of the mesentery including pancreas (labeled as pancreas), intestines, bile and digestive fluids of medaka. An OlPac2 band is present in the mesentery (including pancreas) sample as a 31 kDa protein corresponding to the pro-enzyme form (*M*_w_ = 28,670.23 Da; Fig. 7). OlPac2 is not present in the intestine extract, but yields a band in the digestive fluids as a 22 kDa protein corresponding to the active form (*M*_w_ = 23,234.35 Da). OlPac3 is visible in its pro-enzyme form (*M*_w_ = 27,356.02) as a 28 kDa protein yielding a clear band in the mesentery (including pancreas) sample, and a weak band in the bile sample. The active form of OlPac3 (*M*_w_ = 22,953.22) yields a band around 20 kDa in the digestive fluid sample (Fig. 7). No signals were detected in intestine extract.

### Pactacin activity in digestive fluid of medaka

**Figure 8:**
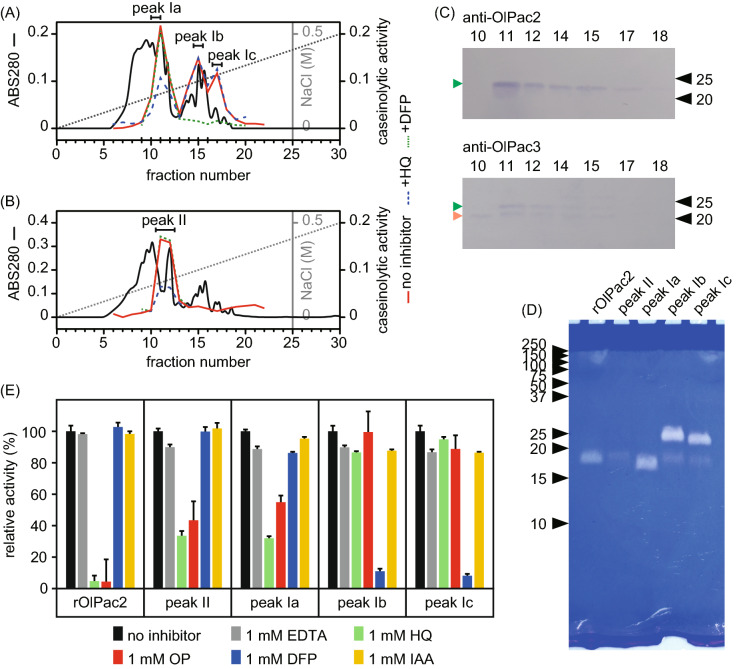
Pactacin activity in the digestive fluid of medaka. Source 15Q chromatogram of untreated (**A**) and DFP-treated digestive fluid (**B**). Black line, protein amount; red line, caseinolytic activity; blue broken line, caseinolytic activity following 1 mM HQ treatment; green dotted line, caseinolytic activity following 1 mM DFP treatment; gray dotted line, NaCl concentration. (**C**) Western blot of (untreated) fractions 10, 11, 12, 14, 15, 17, and 18. Anti-OlPac2 or anti-OlPac3 antibodies were used. Black triangles indicate molecular marker positions (values are indicated in kDa). Green triangles indicate the positive signal from the anti-OlPac2 antibody (upper panel) or the cross reaction of the anti-OlPac3 antibody with OlPac2 (lower panel). The orange triangle indicates the signal from the anti-OlPac3 antibody. Band regions are cropped, and full-length photos are included in a Supplementary Information. (**D**) Casein acrylamide gel zymography of rOlPac2 and peaks Ia, Ib, Ic, and II. Numbers indicate the molecular mass (kDa) of molecular markers. (**E**) Inhibition of caseinolytic activity of rOlPac2 and peaks Ia, Ib, Ic and II. Values (%) indicate activity following treatment with 1 mM EDTA (gray), HQ (green), OP (red), DFP (blue) or IAA (yellow) relative to that of untreated samples.

To further characterize pactacin, we undertook partial purification of pactacin from the digestive fluid of medaka, and also realized the synthesis of a recombinant pactacin. First, we tried to synthesize recombinant OlPac2 and OlPac3; an active enzyme could be obtained only for the former (rOlPac2). The activity of astacin family metalloproteases is known to be inhibited by chelating agents such as 8-hydroxyquinoline-5-sulfonic acid (HQ), ortho-phenanthroline (OP), and ethylenediaminetetraacetic acid (EDTA)^16^. The caseinolytic activity of rOlPac2 was examined using various inhibitors: the metalloprotease inhibitors HQ, OP, and EDTA; a serine protease inhibitor, diisopropyl fluorophosphate (DFP); and a cysteine protease inhibitor, iodoacetamide (IAA). Interestingly, the caseinolytic activity of rOlPac2 was not inhibited by EDTA, but was strongly inhibited by the other metalloprotease inhibitors, HQ and OP (Fig. 8E). The caseinolytic activity was not inhibited by DFP or IAA (Fig. 8E). Therefore, we conclude that rOlPac2 is a metalloprotease, although the inhibitory effect of chelating agents on this enzyme is somewhat different from that observed for other astacin family proteases.

Proteases in the digestive fluid of medaka were separated by anion exchange column chromatography. Digestive fluid is considered to contain several digestive enzymes, the most important ones being the serine proteases trypsin and chymotrypsin, in addition to pactacin. Identification of the pactacin fraction was achieved by incubating part of the digestive fluid with DFP before performing column chromatography.

The caseinolytic activities of the eluted fractions obtained from non-DFP treated digestive fluid are shown in Fig. 8A. Three proteolytic peaks, designated Ia, Ib, and Ic, were detected. The activity of the Ia peak was halved by HQ, but not inhibited by DFP, while peaks Ib and Ic were completely suppressed in the presence of DFP, but were unaffected by HQ. Fractionation of DFP-treated fluid gave a single active peak, designated peak II, with a retention time corresponding to that of peak Ia. As also observed for peak Ia, the activity of peak II was inhibited by HQ (Fig. 8B), but not by EDTA, DFP or IAA (Fig. 8E). The activity of proteases corresponding to peaks Ia and II was qualitatively inhibited in the same way as rOlPac2, even though it was not completely inhibited by HQ or OP, probably because fractionation did not yield perfect separation of proteins. On the other hand, the caseinolytic activity of proteases corresponding to peaks Ib and Ic was only inhibited by DFP. These results suggest that peaks Ia and II contain metalloproteases (probably pactacin) and peaks Ib and Ic correspond to serine proteases (probably trypsin and/or chymotrypsin).

The active fractions (10, 11, 12, 14, 15, 17, and 18) obtained from Source 15Q column chromatography of non-DFP-treated fluid were subjected to SDS-PAGE and western blot analysis using anti-OlPac2/3 antibodies (Fig. 8C). Anti-OlPac2 antibody reacted strongly with the 22 kDa protein in fraction 11, and the signal became weaker from fraction 12 to 17 (Fig. 8C). Anti-OlPac3 antibody weakly reacted with two bands corresponding to the 22 and 20 kDa proteins in fractions 10–12 (Fig. 8C). As described in the section “western blot analysis”, OlPac2 and OlPac3 yield a bands around 20 and 22 kDa, respectively. On the basis of high sequence similarity, we determined that the 20 kDa band corresponded to a specific reaction with OlPac3, and the 20 kDa band was the result of a cross reaction with OlPac2. The band intensities of the fractions were consistent with the elution pattern monitored by caseinolytic activity (Fig. 8A).

Zymographic analysis revealed that the major proteases corresponding to peaks Ia, Ib, and Ic had molecular masses of 18, 25 and 23 kDa, respectively (Fig. 8D). Peaks II and Ia yielded bands of essentially the same mobility as rOlPac2. These results suggest that the major protease in peak Ia is OlPac2.

## Discussion

During the evolution of teleosts, gene duplication occurred and many kinds of astacin metalloproteases appeared^17^. Some of them, such as BMP1 and meprin, are commonly found in vertebrates, while others, referred to as six-cysteine-containing astacin proteases (C6AST), are mainly found in teleosts^3,18,19^.

In this study, we discussed evidence for the function of one of the C6ASTs we named pactacin. Pactacin enzymes can be divided into three subclades, which appeared by gene duplication during teleostean evolution. Although some of the copied genes disappeared in independent fish lineages, genes of the three subclades showed common expression in pancreatic cells, similar to trypsinogen genes. Immunohistochemical analysis revealed that pactacins are synthesized in pancreatic cells and stored in bile as the pro-enzyme form, and then secreted into the intestines in the active forms. These results suggest that pactacin is synthesized in the pancreas and then secreted into the intestines to function as a digestive enzyme.

We fractionated the digestive fluid of medaka and detected three major proteases: a metalloprotease, pactacin, and two serine proteases, trypsin and/or chymotrypsin. The caseinolytic activity of the pactacin peak (peak Ia) is higher than that of the two serine protease peaks (peaks Ib and Ic). The ratios of the caseinolytic activities of the three peak fractions were 5:3:2 (for peaks Ia, Ib, and Ic). These results suggest that pactacin is one of the major digestive enzymes in medaka.

The presence of stomach (gastric glands) is not universal in vertebrates^20^. Stomach-less fishes have been found from various families, such as Atherinopsidae (silversides)^21^, Cyprinodontidae (killifish)^22^, Hemiramphidae (halfbeak), Belonidae (needlefish)^23^, Labridae (ballan wrasse)^24^, Blenniidae (combtooth blennies)^25^, Cyprinidae (carp)^26,27^, Tetraodontidae (puffer fish)^28^, Odacidae (greenbone), Scaridae (parrotfish)^29^, Cobitidae (loaches)^30^, Gobiidae (goby)^31^ and Syngnathidae (seahorse)^27^. Organisms without a stomach are characterized by the deletion of the gastric genes, i.e., H^+^/K^+^-ATPase (Atp4a and Atp4b) and pepsinogen genes^20^. We searched gastric genes in the seahorse genome and were unable to find any orthologs. These results confirmed that the seahorse is also a stomach-less fish. Considering the diverse evolution of the digestive organs in teleosts, it is possible that teleosts developed novel digestive strategies that do not involve pepsins. In cray fish (*Astacus astacus*), astacin was reported to function as a digestive enzyme, and showed low substrate specificity^32^. Since astacin gene orthologs, encoding for digestive enzymes, are not found in vertebrates, it is conceivable that teleost redeveloped novel digestive enzymes from the astacin protease family.

The present phylogenetic analysis clearly indicates that the C6ASTs used in this study can be divided into 3 clades: pactacin, nephrosin and patristacin. As discussed above, three pactacins, from three different subclades, are commonly expressed in pancreatic cells, and function as digestive enzymes. Many nephrosins have been reported to express in the kidney^3,6^. Both pactacin and nephrosin genes show clade-specific expression patterns. In contrast, patristacin genes show diverse expression patterns. The patristacin clade is further divided into 2 subclades. Subclade 2 genes are only found in cyprinodontiform fishes, while subclade 1 genes are found in syngnathiform, cyprinodontiform and perciform fishes. Interestingly, the expression pattern of patristacin subclade 1 genes has been reported not to conserve among the fish species as follows. In pipefish and seahorse (Syngnathiformes), the patristacin gene is expressed in the brood pouch^8,12^. From cichlid (a perciform fish), a patristacin gene called cimp1 was reported to be expressed in the gills and jaws^33^. As distinct expression sites are found for patristacin genes in different species, it appears that this clade modulates diverse protein functions. To identify the function(s) of patrsitacin, it is necessary to determine its exact expression tissues or cell types in the brood pouch of pipefish and seahorse, and in the gills and jaws of cyprinodontiform fishes.

## Materials and methods

### Animals

Adult orange-red variants of medaka and adult pot-bellied seahorses were purchased from a local dealer and kept in indoor tanks at 27 °C and 20 °C, respectively. Mice (Jcl:ICR) were bred under specific-pathogen-free conditions. Genetic experimental protocols were approved by the Genetic Modification Experiment Safety Committee of Sophia University, Tokyo, Japan.

### Cloning of seahorse C6AST cDNAs

First-strand cDNA was synthesized from RNA extracted from the liver, kidney, and brood pouch of a mature male seahorse using a SMART™ RACE cDNA Amplification Kit (Clontech Laboratories, Mountain View, CA, USA). cDNA fragments of the C6AST genes were amplified using degenerate primers (F1–4 and R1) designed from conserved regions of astacin metalloproteases, as described previously^34^. Gene-specific primers were designed from the cloned fragments and used for 5′ RACE and 3′-RACE PCR.

### Phylogenetic analysis

We searched for homologous genes of nephrosin (HaNpsn), pactacin (HaPac1), and patristacin (HaPastn) with the GenBank BLAST program, while the genome data was used to search for gene homologs to those of tiger tail seahorse (LVHJ00000000.1) and greater pipefish (*Syngnathus acus*; CABFKE000000000.2). Novel sequences not registered in GenBank were isolated by in silico cloning of the region homologous with that of pot-bellied seahorse according to the GT-AG rule. In total, 105 genes were used in the phylogenetic analysis. Amino acid sequences of the astacin protease domain were aligned using the ClustalX program. Maximum-likelihood (ML) analysis, using a WAG model with I + Γ, was conducted with RAxML version 8.2.10^35^. To determine the best-scoring topology, the tree was reconstructed with 1000 bootstrap replicates.

### Semi-quantitative RT-PCR expression analysis

One mature male seahorse was dissected to isolate the hepatopancreas, gills, heart, kidney, mesentery including pancreas, intestines, and brood pouch. From mature female and male medaka, the gills, heart, kidney, spleen, liver, mesentery including pancreas, intestines, ovaries and testes were isolated. RNA sequences were extracted using RNAiso (Takara Bio Inc., Otsu, Japan) according to the manufacturer’s instructions, and were used to synthesize cDNA with PrimeScript Reverse Transcriptase (Takara Bio Inc.) and a poly-dT primer. The cDNA fragments were amplified with EmeraldAmp^®^ PCR Master Mix (Takara Bio Inc.) using the primer sets shown below. The cDNA amounts added to the PCR cocktail were normalized by the intensity of the amplified β-actin band.

- Seahorse HaPac1-F: 5′-TGTTGTTTTCTGCCTACTGTTGG-3′
- Seahorse Ha Pac1-R: 5′-CGATGCTCAGCCAGTCACGGTCA-3′
- Seahorse trypsinogen-F: 5′-CTGGTCTTCATTCTGCTCATCGG-3′
- Seahorse trypsinogen-R: 5′-CAGCTTGTTCTTGTCGGCGGTGG-3′
- Medaka OlPac2-F: 5′-CAATCCGTTCTGAGGCCGACAAG-3′
- Medaka OlPac2-R: 5′-CAATATCATTCTTGCTCATCTGG-3′
- Medaka OlPac3-F: 5′-GTGTACATACCCTATGTGATTGC-3′
- Medaka OlPac3-R: 5′-AAGTGTTGAGTTTATCAAAGGCG-3′
- Medaka trypsinogen-F: 5′-CAGTTACAATTCCTACACCATTG-3′
- Medaka trypsinogen-R: 5′-CCAGGTATCTTAGGTTGAATCAC-3′
- β-actin-F: 5′-ACACCTTCTACAACGAGCTG-3′
- β-actin-R: 5′-GTACAGGTCCTTACGGATGT-3′

### In situ hybridization

The abdominal part including liver, pancreas, intestines, and kidney from adult seahorses was dissected and fixed in a 4% paraformaldehyde phosphate buffer (PB; pH 7.4). The sample was transferred to a 30% sucrose solution in phosphate buffered saline (PBS; pH 7.4), embedded in Optimal Cutting Temperature (OCT) Compound (Sakura Finetek, Tokyo, Japan), and cut into 10-μm-thick sections using cryostat (CryoStar NX50, Thermo Fisher Scientific). The sections were immersed in a hybridization buffer containing 50% formamide, 5 × SSC (pH 6.0), 0.1% Tween-20, 50 μg/mL tRNA and 50 μg/mL heparin for 2 h at 55 °C, and then hybridized overnight in the hybridization buffer containing the RNA probe. The slides were then washed three times for 30 min in 50% formamide and 2 × SSC (pH 6.0) containing 0.1% Tween-20 (SSCT) at 68 °C, four times for 15 min in 2 × SSCT at 68 °C, three times for 20 min in 0.2 × SSCT at 68 °C, and three times for 5 min in PBS containing 0.1% Tween-20 (PBST) at room temperature. The sections were blocked for 90 min with 1% blocking reagent in PBST, and then incubated with 1:8000-diluted alkaline phosphatase–conjugated anti-digoxigenin antibody in PBST at 4 °C overnight. After six 30-min washes in PBST, the sections were pre-equilibrated for 5 min in a staining buffer (pH 9.5) consisting of 100 mM Tris–HCl, 50 mM MgCl_2_, 100 mM NaCl and 0.1% Tween-20, and then stained with 1:50 (v/v) NBT/BCIP added to the staining buffer.

### Antibody preparation

Recombinant proteins of seahorse HaPac1 and medaka OlPac2 and OlPac3 were prepared as described previously^36,37^. Briefly, cDNA fragments containing the astacin protease domain were amplified by PCR. The amplified fragment was ligated into the pET3c expression vector. The recombinant pactacin was expressed in *E. coli (BL21)*, and purified as the inclusion body. Then, the inclusion body was dissolved in 50 mM Tris–HCl (pH 8.0), 8 M urea, 0.1 M 2-mercaptoethanol and 1 mM EDTA, and then dialyzed in 8 M urea. These proteins were used as antigens to raise anti-pactacin antibodies in mice. Four antigen injections were performed, followed by extraction of serum for isolation of antibodies.

### Immunohistochemistry

The abdominal part including liver, pancreas, intestines, and kidney of seahorse and medaka was dissected and fixed in 4% paraformaldehyde. After fixation, the samples were washed with PBS, dehydrated in graded ethanol solutions (50, 70, 90, 95 and 100% v/v in water), and embedded in paraffin. Immunohistochemistry and hematoxylin–eosin (HE) staining were performed on histological (4–8-μm thick) sections as described previously^38^. Briefly, after the sections were blocked with 2% bovine serum albumin (BSA) in TBST (50 mM Tris–HCl, 150 mM NaCl, 0.05% Tween-20) at 4 °C overnight, the sections were incubated with the primary antibody diluted 100-fold in the blocking buffer at room temperature for 2 h. After rinsing the sections three times for 5 min with TBST, they were incubated with the secondary (alkaline phosphatase–conjugated anti-mouse IgG) antibody, diluted 1:2000 in TBST, for 2 h. Sections were rinsed five times for 5 min in TBST, pre-equilibrated in 100 mM Tris–HCl (pH 9.5) for 5 min, and developed with NBT/BCIP.

### Western blotting

Tissue samples of medaka mesentery, including the pancreas, were homogenized in PBS and centrifuged at 20,000*g* for 10 min to obtain supernatant fractions. The intestines from medaka was isolated and washed by pouring PBS into the gut. Intestine contents were thus collected in PBS. The gallbladder was isolated and perforated, and the bile was collected by draining. Western blot analysis was performed with the anti-OlPac2 or anti-OlPac3 antibodies. The proteins were transferred to a poly vinylidene di-fluoride (PVDF) membrane after separation by SDS-PAGE. The membrane was blocked with 2% BSA in TBST at 4 °C overnight, followed by incubation with the primary antibody diluted in the blocking buffer (1:500 for OlPac2 and OlPac3) at room temperature for 2 h. After rinsing the membrane three times for 5 min with TBST, proteins were incubated with the secondary antibody, diluted 1:2000 in TBST, for 2 h. The membrane was rinsed five times for 5 min TBST, pre-equilibrated in 100 mM Tris–HCl pH 9.5 for 5 min, and developed with NBT/BCIP.

### Inclusion body refolding

Inclusion bodies of OlPac2 and OlPac3 were dissolved in 50 mM Tris–HCl (pH 8.0), 8 M urea, 0.1 M 2-mercaptoethanol and 1 mM EDTA at 37 °C for 20 min. After centrifugation at 20,000*g* for 10 min, the supernatant was diluted in 8 M urea to a final protein concentration of 0.4 mg/mL. The protein solution was dialyzed against refolding buffer (50 mM Tris–HCl (pH 8.0), 0.8 M l(+)-arginine hydrochloride, 0.5 mM glutathione, 0.1 mM oxidized glutathione and 5 μM ZnSO_4_) at 4 °C for 2 days, and against 25 mM Tris–HCl (pH 8.0; once with 5 μM ZnSO_4_ and twice without ZnSO_4_) at 4 °C. The folding mixture was centrifuged at 20,000*g* for 10 min, concentrated using Amicon Ultra 10 K filter units (Millipore, Billerica, MA), and the supernatant was used as the active enzyme.

### Partial purification of pactacin

The intestines from 20 adult medaka were isolated, and the digestive fluid was washed by injecting 200 μL of 10 mM Tris–HCl buffer (pH 7.5) twice into the gut. The digestive fluid thus collected was centrifuged at 15,000*g* for 5 min, and the supernatant was filtered using a 0.45-μm membrane. Next, one part of the fluid was directly applied to a Source 15Q (GE Healthcare Life Science, USA) column mounted in an HPLC system (Cilson, Middleton, WI, USA). The remaining fluid was incubated with 1 mM DFP at 30 °C for 15 min prior to injection into the column. After the column was washed with a 10 mM Tris–HCl buffer (pH 7.5), the adsorbed protein was eluted with a 10 mM Tris–HCl buffer (pH 7.5) employing a linear gradient (0–0.5 M) of NaCl concentration and a constant flow rate of 1 mL/min. Eluted fractions were collected for one minute each.

### Caseinolytic activity and protease inhibition

Using 10 μL of the eluted fractions, the caseinolytic activity was measured from their reaction with a buffered casein solution (375 μL, 3.3 mg/mL casein, 83 mM Tris–HCl, pH 7.5)^39^. The mixture was incubated for 30 min at 30 °C. After the reaction was stopped by adding 125 μL of 20% perchloric acid, the reaction mixture was allowed to stand in an ice-cold water bath for 10 min, and centrifuged at 20,000*g* for 5 min at 10 °C. The absorbance at 280 nm of the supernatant was measured.

For inhibition studies, eluted fractions (10 μL) were incubated with an inhibitor (1 mM of HQ, OP, EDTA, DFP, or IAA) solution for 10 min at 30 °C before testing caseinolytic activity as described above.

### Zymography

Different fractions (peaks Ia, Ib, Ic, and II, and the synthesized rOlPac2) were mixed with SDS-PAGE buffer (2% SDS, 10% glycerol, and 0.01% bromophenol blue, 62.5 mM Tris–HCl pH6.8) without 2-mercaptoethanol, and incubated for 30 min at 80 °C. Electrophoresis of the mixture was run on a 15% polyacrylamide gel containing 0.1% casein. The gel was subsequently rinsed twice with 2% Triton X-100 in 20 mM Tris–HCl pH 7.5 buffer for 30 min, and once with 1% Triton X-100 in 50 mM Tris–HCl pH 8.0 buffer for 30 min. The gel was then incubated in 50 mM Tris–HCl pH 8.0 buffer containing 1 μM ZnSO_4_ for 2 h at 30 °C. Bands of casein degradation were visualized by staining the gels with Coomassie brilliant blue in methanol/acetic acid mixture overnight, followed by rinsing in water.

### Ethics approval

Animal care and animal experiments were done in accordance with the guidelines of the Animal Experiment Committee of Sophia University, Tokyo, Japan.

## Supplementary Information

Supplementary Information.

## Data Availability

The nucleotide sequence data reported here will appear in the DDBJ/EMBL/GenBank databases under accession numbers LC602897, LC602898, and LC602899.
