# Supplementary material for: Pactacin is a novel digestive enzyme in teleosts
Source: Sci Rep. 2021 Mar 31;11:7230. doi: 10.1038/s41598-021-86565-9 (PMC8012361; doi:10.1038/s41598-021-86565-9)
Supplement: Supplementary file 1 — Supplementary Information. [file 41598_2021_86565_MOESM1_ESM.pdf]

Pactacin is a novel digestive enzyme in teleosts

Mari Kawaguchi, Yohei Okazawa, Aiko Imafuku, Yuko Nakano, Risa Shimizu, Reiji Ishizuka, Tianlong Jiang, Tatsuki Nagasawa, Junya Hiroi, and Shigeki Yasumasu

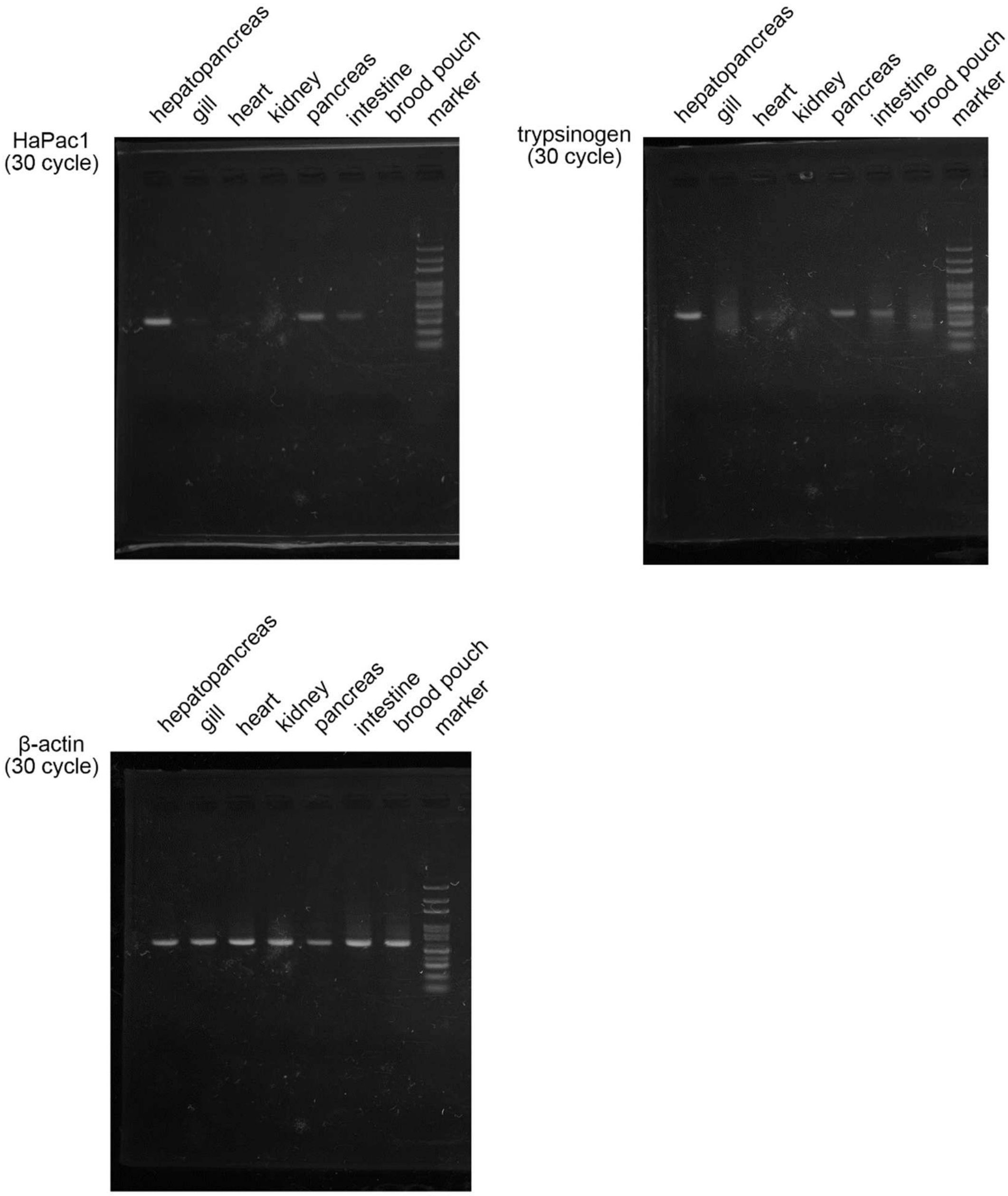

Supplementary Figure 1 Full-length gel images of semi-quantitative expression analysis. HaPac1 and trypsinogen gene expression in adult seahorse hepatopancreas, gills, heart, kidney, mesentery including pancreas (labeled as pancreas), intestines, and brood pouch.β-actin was used as a control signal.

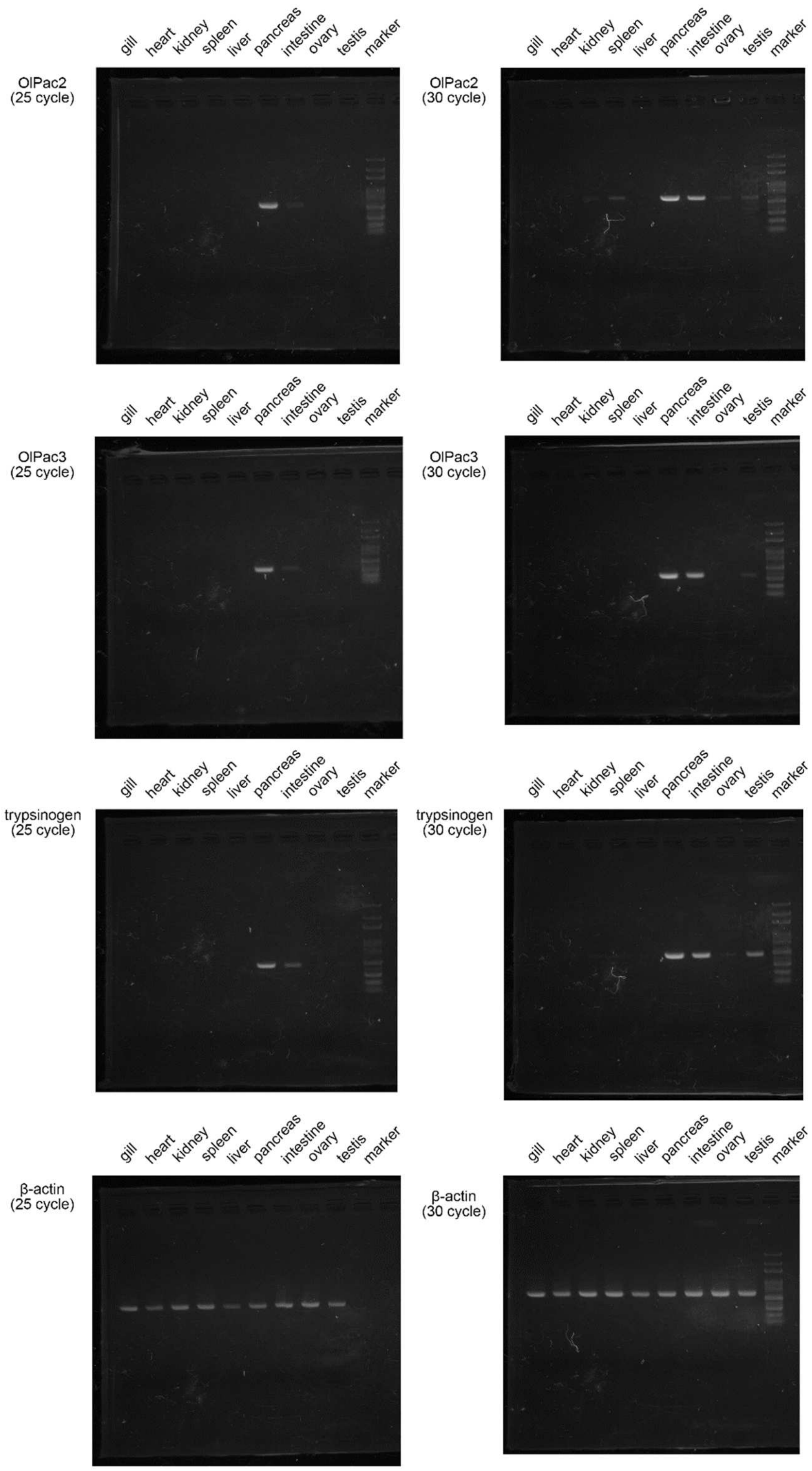

Supplementary Figure 2 Full-length gel images of semi-quantitative expression analysis. OlPac2 (MC6AST2), OlPac3 (MC6AST3), and trypsinogen gene expression in adult medaka gills, heart, kidney, spleen, liver, mesentery including pancreas (labeled as pancreas), intestines, ovary and testes. β-actin was used as a control signal.

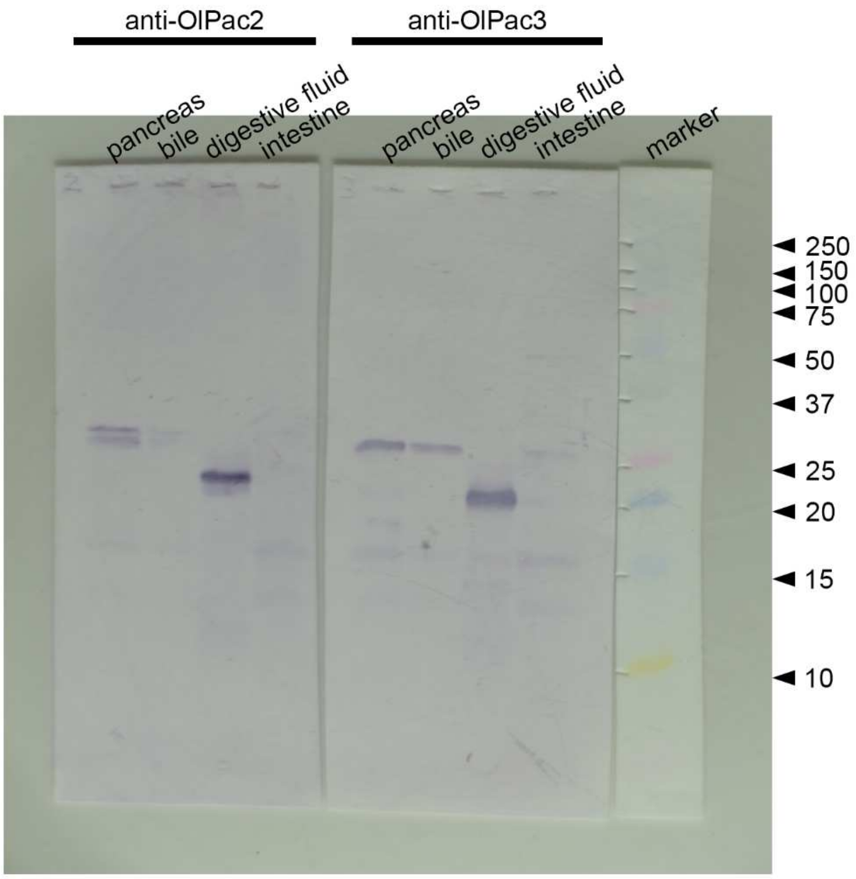

Supplementary Figure 3 Original photo of western blot analysis of pactacin. Medaka extracts of the mesentery including pancreas (labeled as pancreas), intestines, bile and digestive fluid with anti-OlPac2 antibody or anti-OlPac3 antibody. Numbers on the left refer to molecular weights (kDa) of molecular markers.

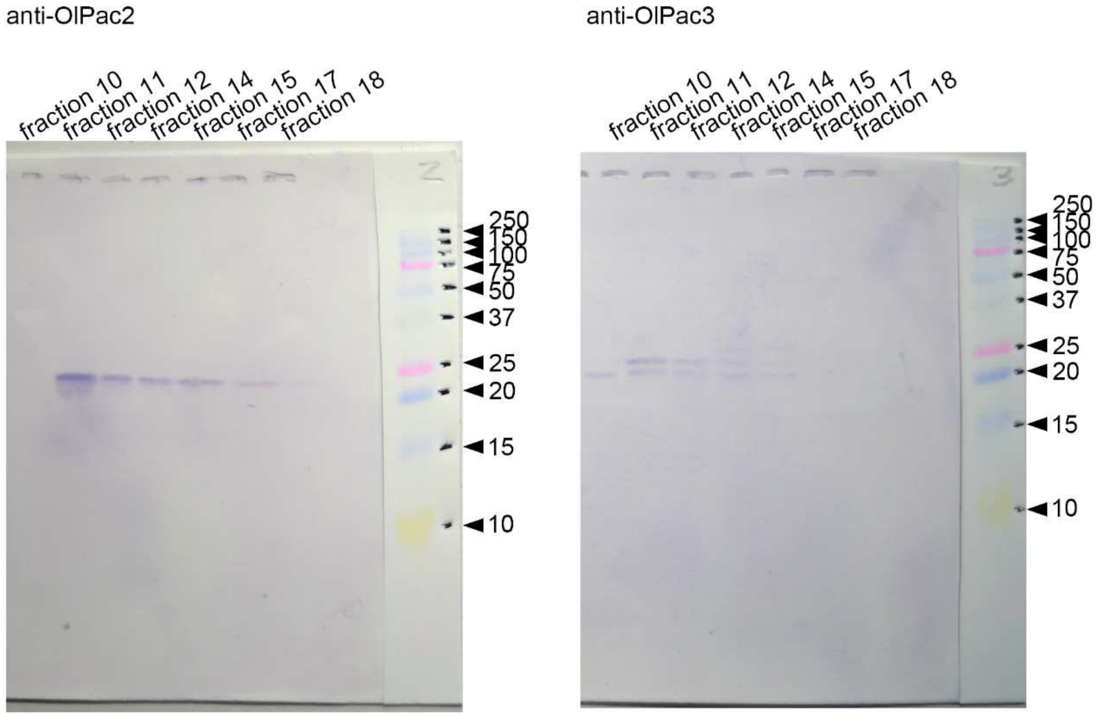

Supplementary Figure 4 Original photo of western blot analysis of fractions 10, 11, 12, 14, 15, 17 and 18. Anti-OlPac2 or anti-OlPac3 antibodies were used. Numbers on the left refer to molecular weights (kDa) of molecular markers.
